# Supplementary material for: Long term term follow-up of tyrosine kinase inhibitors treatments in inoperable or relapsing diffuse type tenosynovial giant cell tumors (dTGCT)
Source: PLoS One. 2020 May 20;15(5):e0233046. doi: 10.1371/journal.pone.0233046 (PMC7239463; doi:10.1371/journal.pone.0233046)
Supplement: S1 File — (DOCX) [file pone.0233046.s001.docx]

Supplementary information : unidentified data of patients included in this analysis
