# Supplementary material for: Long term term follow-up of tyrosine kinase inhibitors treatments in inoperable or relapsing diffuse type tenosynovial giant cell tumors (dTGCT)
Source: PLoS One. 2020 May 20;15(5):e0233046. doi: 10.1371/journal.pone.0233046 (PMC7239463; doi:10.1371/journal.pone.0233046)
Supplement: Attachment — Submitted filename: Responses.docx [file pone.0233046.s002.docx]

Prof Jean-Yves BLAY

Medical Oncology Department

General Director

Centre Léon Bérard,

28, rue Laënnec,

69373 Lyon Cedex 08,

France

[jean-yves.blay@lyon.unicancer.fr](mailto:jean-yves.blay@lyon.unicancer.fr)

*PLoS One*

Editorial office

Lyon, March 25th 2020

Reference: PONE-D-19-26498R2

Long term term follow-up of tyrosine kinase inhibitors treatments in inoperable or relapsing diffuse-type tenosynovial giant cell tumors (dTGCT).

PLOS ONE

Dear Editor,

I am sorry, I realized I put the previous version of the Ms on the site yesterday.

We have added in the newly revised version

1) the reference of the grants and the the grantees (in the final version of the Ms without track changes),
2) the first sentence related to the declaration of interest (in the final version of the Ms without track changes),
3) and the date and addresses of the IRB of the Centre Leon Berard (on the website of PLoS One and in the material and method section)
4) Anonymised patient data (no names, not dates, no identifier) are added as supplementary information, in an additional file added to this R3 version.

We remain at your disposition for any further questions you may have on this work.

With kind regards,

Prof. JY Blay
